# Case Report: Correlation between pulmonary capillary wedge pressure and left-ventricular diastolic pressure during treatment with veno-arterial extracorporeal membrane oxygenation

**DOI:** 10.3389/fcvm.2023.1271227

**Published:** 2023-10-23

**Authors:** Rajat Kalra, Christopher Gaisendrees, Tamas Alexy, Marinos Kosmopoulos, Deborah Jaeger, Georg Schlachtenberger, Ganesh Raveendran, Jason A. Bartos, Alejandra Gutierrez Bernal, Ranjit John, Thorsten Wahlers, Demetris Yannopoulos

**Affiliations:** ^1^ Cardiovascular Division, University of Minnesota, Minneapolis, MN, United States; ^2^ Center for Resuscitation Medicine, University of Minnesota, Minneapolis, MN, United States; ^3^ Department of Cardiothoracic Surgery, University Hospital of Cologne, Cologne, Germany; ^4^ INSERM U 1116, University of Lorraine, Vandoeuvre-lès-Nancy, France; ^5^ Cardiothoracic Surgery Division, University of Minnesota, Minneapolis, MN, United States

**Keywords:** VA-ECMO, ECLS (VA), PCWP, pulmonary capillary wedge pressure, LVEDP, left ventricular end-diastolic pressure

## Abstract

**Background:**

Pulmonary capillary wedge pressure (PCWP) is often used as a surrogate for left-ventricular end-diastolic pressure in patients (LVEDP) who are on veno-arterial extracorporeal membrane oxygenation (V-A ECMO) support for cardiogenic shock and cardiac arrest. However, the correlation between PCWP and LVEDP is not clear in the setting of V-A ECMO usage. We sought to evaluate this correlation in this case series.

**Methods:**

Patients were referred to our cardiac catheterization laboratory for invasive hemodynamic studies to assess their readiness for VA-ECMO decannulation. All patients underwent simultaneous left and right heart catheterization. Using standard techniques, we measured PCWP and LVEDP simultaneously. Continuous variables were reported as medians with interquartile ranges. The correlation between PCWP and LVEDP was evaluated using simple linear regression and reported as *R*^2^.

**Results:**

Four patients underwent invasive hemodynamic studies 4 (2.5, 7) days after VA-ECMO cannulation. All four patients had suffered in-hospital cardiac arrest and had been put on VA-ECMO. At the baseline level of VA-ECMO flow of 4.1 (3.8, 4.4) L/min, the median LVEDP and PCWP were 6 (4, 7.5) mmHg and 12 (6.5, 16) mmHg, respectively. At the lowest level of VA-ECMO flow of 1.9 (1.6, 2.0) L/min, the median LVEDP and PCWP was 13.5 (8.5, 16) mmHg and 15 (13, 18) mmHg, respectively. There was a poor correlation between the simultaneously measured PCWP and LVEDP (*R*^2 ^= 0.03, *p* = 0.66).

**Conclusions:**

The PCWP may not correlate well with LVEDP in patients treated with VA-ECMO, particularly at high levels of VA-ECMO support.

## Introduction

Extracorporeal cardiopulmonary resuscitation (ECPR) with veno-arterial extracorporeal membrane oxygenation (V-A ECMO) is a strategy that has produced improvement in mortality in out-of-hospital cardiac arrest (1, 2). With the publication of recent trials showing the benefit of this strategy (3–6), it is likely that V-A ECMO will be subject to wider adoption worldwide as part of ECPR strategies.

Despite the demonstrated utility of the ECPR strategy, there is still significant debate about the effect of V-A ECMO on intracardiac hemodynamics in cardiogenic shock and cardiac arrest. Pulmonary capillary wedge pressure (PCWP) is often used as a metric of preload. This is under the assumption that PCWP reflects left atrial pressure and/or left-ventricular end-diastolic pressure (LVEDP) (7). Additionally, right heart catheterization is used in patients on V-A ECMO to evaluate intracardiac hemodynamics during the course of V-A ECMO treatment. While these principles may apply to patients that do not have mechanical support strategies in place, the utility of PCWP and its correlation to LVEDP has not yet been comprehensively evaluated in patients with V-A ECMO.

In this case series, we evaluated the relationship of PCWP with LVEDP with simultaneous left and right heart catheterization in four patients treated with V-A ECMO as part of an ECPR strategy. We sought to evaluate whether the PCWP and LVEDP varied with changes in V-A ECMO flow, the direction in which they changed with changes in V-A ECMO flow, and the degree of correlation between these two metrics.

## Methods

### Study population

All patients were evaluated between November 2021 and June 2022. All four patients were placed on V-A ECMO and enrolled in our local ECPR protocol after in-hospital cardiac arrest or as part of the Minnesota Resuscitation Consortium's previously published protocol (8) for out-of-hospital cardiac arrest. All patients had cannulation with the venous and arterial cannulae in the femoral vasculature (V_f_-A_f_ ECMO). Their intensive care also followed a protocolized pathway that we have published previously (9). All hemodynamic data were collected prospectively. The study was approved by our institution's Institutional Review Board (study number 00005355).

### Inclusion criteria

The four subjects described in this case series were referred for invasive hemodynamic case series as part of routine clinical care. The invasive hemodynamic studies were sought due to marginal recovery of the patient's cardiac function that led the referring critical care physician(s) to be uncertain about the patients' candidacy for V-A ECMO decannulation. This led the cardiac critical care team to invasively evaluate changes in LV and RV. All subjects were treated with aggressive supportive measures and volume removal prior to the invasive hemodynamic studies, and the timing of the invasive hemodynamic study was left to the treating clinician's judgement.

### Invasive hemodynamic turndown study protocol

Our institution's echocardiographic turndown (weaning) protocol has been published previously (10). In this protocol, the V-A ECMO support is sequentially reduced from the baseline flow to 1–2 L/min while a methodical evaluation of LV and RV function is obtained. The reductions in V-A ECMO flow are done in 1 L/min increments. At each stage, the V-A ECMO flow is maintained for at least three minutes and hemodynamic stability (as measured by aortic mean arterial pressure >60 mmHg) is ensured before further decreasing the level of support. At the lowest level of support, the flow is maintained for at least five minutes to allow for a steady state to be achieved. Patients are only referred for turndown studies if they are on low or moderate doses of ≤2 vasopressors/inotropes.

In addition to our echocardiographic turndown data, we adapted our turndown protocol to include measurements of left and right heart pressures at the baseline (highest) and lowest V-A ECMO flow. Left ventricular pressures were measured by obtaining left radial access under ultrasound and fluoroscopy guidance using a 6 Fr slender sheath. A 6 Fr multipurpose diagnostic catheter was then inserted via the sheath into the aortic root. After this, an Opsens Optowire pressure wire was positioned in the LV cavity. Dual-lumen pigtail catheters were not available in our laboratory during the study period (11). All patients had right heart (Swan-Ganz) catheters inserted via the right internal jugular vein after this under ultrasound and fluoroscopic guidance. We then measured aortic, LV end-diastolic, right ventricular (RV) end-diastolic, pulmonary artery systolic, pulmonary artery diastolic, and pulmonary capillary wedge pressures in all four patients at the baseline and lowest tolerated V-A ECMO flows as part of the above turndown protocol. The RVEDP was reported as a measure of right-sided filling pressures. Right atrial pressure was not measured due to the fact that the venous VA-ECMO cannula was in the right atrium in the patients and it was assumed that the measurements would be subject to significant artifact. The left and right heart catheters were zeroed at each phase of the V-A ECMO flow. None of the hemodynamic measurements were obtained with any unloading strategy (intra-aortic balloon pump, percutaneous ventricular assist device, or left atrial venoarterial cannulation) in place. All pressure measurements were taken at the trough of the respiratory cycle. Since all patients were mechanically ventilated at the time of the examination, this was done to ensure that the pressure measurements were taken at end-expiration (12). Catheter placement was confirmed to be in West Zone 3 by ensuring that the PCWP did not change significantly with positive end-expiratory pressure. All invasive hemodynamic measurements were evaluated by two Cardiologists. The first Cardiologist measured them during the procedure and saved the hemodynamic waveforms for offline review. The second Cardiologist evaluated the pressure measurements offline from the saved waveforms and was blinded to the original measurements.

### Statistical analyses

All continuous variables were represented as medians with interquartile ranges. Simple linear regression was done to evaluate the relationship between the LVEDP and PCWP at the baseline level of flow and the LVEDP and PCWP at the lowest level of flow. Results were reported as *R*^2^. The level of significance was 0.05 for all analyses. All analyses were done in Stata/MP 17.0.

## Results

### Baseline characteristics

Four patients with a median age of 54 (46,61) years with SCAI E cardiogenic shock were referred to us for invasive hemodynamic evaluations due to uncertainty regarding their candidacy for VA-ECMO decannulation. Their clinical history is outlined in Table 1.

**Table 1:** Case histories of included patients.

Patient	Case history
1	59-year old male with an in-hospital pulseless electrical asystole arrest due to a hemodynamically unstable (massive) pulmonary embolism. He had an unwitnessed cardiac arrest in a psychiatric unit within our hospital. His initial presenting rhythm was pulseless electrical asystole and he received bystander CPR for 20 min, after which he was transferred to the cardiac catheterization laboratory for V-A ECMO cannulation. He underwent percutaneous pulmonary thrombectomy afterwards. His persistent severe RV dysfunction after the thrombectomy prompted the invasive hemodynamic study. He was decannulated eight days after admission and discharged 42 days after admission. The patient was alive at six months.
2	48-year old male with an in-hospital cardiac arrest due to inferior ST-elevation myocardial infarction. The patient had percutaneous revascularization of the right and left circumflex coronary arteries but then had a pulseless electrical asystole arrest in the intensive care unit approximately 18 h later. He required approximately 10 min of advanced cardiac life support, after which he was immediately transferred to our cardiac catheterization laboratory and placed on V-A ECMO. He had persistent, severe biventricular dysfunction that prompted the invasive hemodynamic study. He was decannulated five days after being placed on V-A ECMO and discharged 39 days after admission. The patient was alive at six months.
3	44-year old female with an in-hospital cardiac arrest due to RV failure five days after liver transplantation for alcoholic cirrhosis. The patient had pulseless electrical asystole and received ACLS for approximately 30 min before undergoing V-A ECMO cannulation. The patient as referred for invasive hemodynamic study due to severe RV failure prior to V-A ECMO cannulation that did not improve on non-invasive turndown studies during her V-A ECMO treatment course. She was decannulated nine days after V-A ECMO placement and discharged 81 days after admission. The patient was alive at six months.
4	62-year old female with an in-hospital cardiac arrest due to shockable rhythm from ST-elevation myocardial infarction. Shortly after the patient underwent emergent coronary angiography and had percutaneous coronary intervention of her proximal left anterior descending artery occlusion, she suffered cardiac arrest due to pulseless ventricular tachycardia and was placed on V-A ECMO. She had persistent severe biventricular dysfunction prompting invasive hemodynamic study. The patient was decannulated six days after V-A ECMO placement but expired in the hospital 47 days after admission due to pulseless electrical asystole due to an unknown cause.

CPR, cardiopulmonary resuscitation; VA-ECMO, veno-arterial extracorporeal membrane oxygenation.

All four patients were placed on V-A ECMO as part of an ECPR strategy after suffering in-hospital cardiac arrest. Three of the patients had pulseless electrical asystole and one patient had pulseless ventricular tachycardia. None of the patients had return of spontaneous circulation prior to V-A ECMO cannulation.

### Invasive hemodynamic study

**Figure 1:**
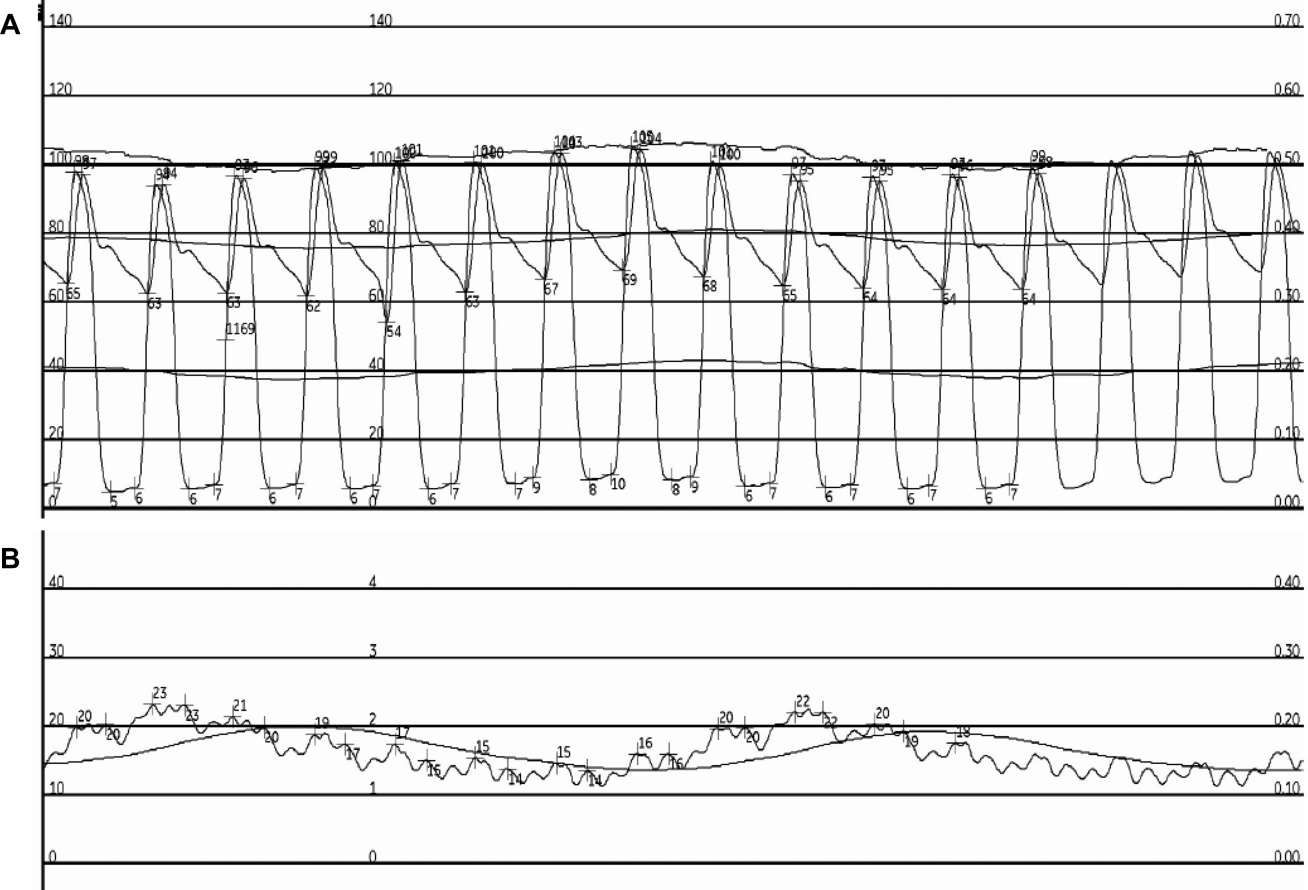
Example pressure tracings from patient 1. Pressure measurements from patient 1 at high V-A ECMO flow are seen. Panel **A** depicts the simultaneous LV and aortic pressure tracings. Panel **B** depicts the patient's PCWP pressure tracing.

All four patients underwent invasive hemodynamic studies due to the treating clinicians' concerning regarding the patients' ability to be decannulated from V-A ECMO. Two patients were referred for the invasive hemodynamic study due to severe biventricular dysfunction on an echocardiogram within 24 h of the invasive hemodynamic study. The two other patients were referred for the invasive hemodynamic study due to severe RV dysfunction on an echocardiogram within 24 h of the invasive hemodynamic study despite supportive therapies. Two of the four patients required low or moderate doses of vasopressors prior to the invasive hemodynamics study. An example of the obtained pressure tracings is seen in Figure 1.

All patients had Doppler evaluation of the mitral and tricuspid valves prior to their invasive hemodynamic study during the same hospitalization. None of the patients had mitral stenosis or tricuspid stenosis.

The median time to the invasive hemodynamic study was 4.0 (2.5, 7.0) days after V-A ECMO cannulation. During the invasive hemodynamic study, the median baseline V-A ECMO flow was 4.1 (3.8, 4.4) L/min and the median lowest V-A ECMO flow was 1.9 (1.6, 2.0) L/min. None of the patients required any adjustments to their ventilatory/oxygenation settings on their mechanical ventilators during the course of the invasive hemodynamic study. Moreover, none of the patients had any changes in the level of inotropic/vasopressive support that they required.

### Hemodynamic evaluation

The PCWP and LVEDP were simultaneously evaluated in all four patients in the cardiac catheterization suite. The PCWP and LVEDP are listed in Table 2 for each patient at the baseline and lowest tolerated V-A ECMO flow. Among all measurements in all four patients, the median LVEDP was 7.5 (5.0, 13.5) mmHg and the median PCWP was 13.5 (11.5, 17.5) mmHg. At the baseline level of VA-ECMO flow, the median LVEDP was 6.0 (4.0, 7.5) mmHg, the median RVEDP was 9.0 (5.0, 14.0) mmHg, and the median PCWP was 12 (6.5, 16.0) mmHg. At the lowest level of VA-ECMO flow, the median LVEDP was 13.5 (8.5, 16.0) mmHg, the median RVEDP was 14.0 (9.5, 22.0) mmHg, and the median PCWP was 15.0 (13.0, 18.0) mmHg.

**Table 2:** Hemodynamic data of included patients.

Patient	V-A ECMO flow (L/min)	Vasopressor usage	Mechanical ventilation parameters	Mean arterial pressure (mmHg)	Heart rate (BPM)	LVEF (percentage)	LVEDP (mmHg)	RVEDP (mmHg)	PA (mmHg)	PCWP (mmHg)
1	Baseline: 3.8	None	RR 12/min, Vt 550 ml, FiO2 40%, PEEP 7 mmHg	78	96	66	8	11	35/18	13
	Lowest: 1.5	None	Unchanged	77	98	77	15	18	43/20	20
2	Baseline: 4.5	None	RR 12/min, Vt 400 ml, FiO2 40%, PEEP 8 mmHg	82	102	16	5	3	12/5	2
	Lowest: 2.0	None	Unchanged	75	107	32	17	10	18/12	12
3	Baseline: 4.3	Norepinephrine 0.15 μg/kg/min, vasopressin 2 units/h	RR 12/min, Vt 400 ml, FiO2 70%, PEEP 8 mmHg	69	110	75	3	17	86/30	19
	Lowest: 1.7	Norepinephrine 0.15 μg/kg/min, vasopressin 2 units/h	Unchanged	81	119	72	5	26	95/40	16
4	Baseline: 3.8	Epinephrine 0.04 μg/kg/min	RR 14/min, Vt 400 ml, FiO2 40%, PEEP 10 mmHg	85	72	10	7	7	18/11	11
	Lowest: 2.0	Epinephrine 0.04 μg/kg/min	Unchanged	74	82	15	12	9	27/15	14

BPM, beats per minute; FiO2, fraction of inspired oxygen; L/min, liters per minute; LVEDP, left ventricular end-diastolic pressure; LVEF, left ventricular ejection fraction; PCWP, pulmonary capillary wedge pressure; PEEP, positive end-expiratory pressure; RR, respiratory rate; V-A ECMO, veno-arterial extracorporeal membrane oxygenation; Vt, tidal volume.

### Correlation between invasive PCWP and LVEDP

**Figure 2:**
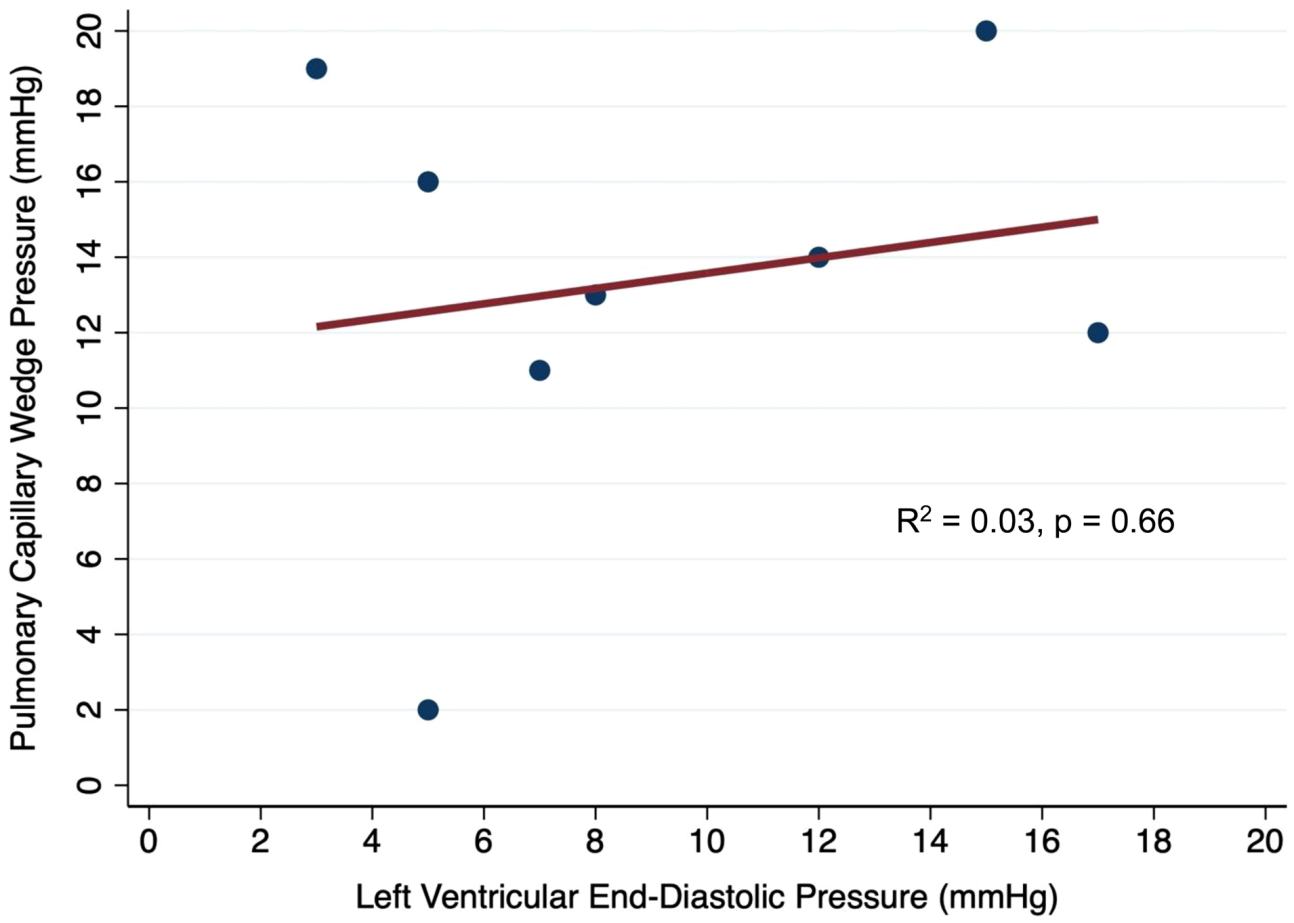
Correlation between simultaneously measured pulmonary capillary wedge pressure and left ventricular end-diastolic pressure in patients on V-A ECMO.

In simple linear regression, there was a poor correlation between the simultaneously measured LVEDP and PCWP (*R*^2 ^= 0.03, *p* = 0.66; Figure 2). When the baseline and lowest levels of V-A ECMO flow were evaluated separately, there was a poor correlation between LVEDP and PCWP that were simultaneously measured at the baseline VA-ECMO flow (*R*^2 ^= 0.05, *p* = 0.77) and the lowest tolerated VA-ECMO flow (*R*^2 ^= 0.02, *p* = 0.86).

## Discussion

In this manuscript, we report the relationship between invasively measured PCWP and LVEDP at two different stages of V-A ECMO flow. In the four patients that received V-A ECMO support as part of an ECPR strategy, we found that there was a poor correlation between the PCWP and LVEDP. Not only did the simultaneously measured values of LVEDP and PCWP exhibit some variation, the direction in which the numbers changed with alterations in V-A ECMO flow also varied among the four patients.

There may be several explanations for our findings. Firstly, V-A ECMO is placed in our institution by inserting the venous cannula via a femoral approach into the right atrium or at the junction of the inferior vena cava and right atrium. This type of cannula placement, which is relatively commonly reported in the literature, is responsible for reducing the amount of venous return to the pulmonary circulation to very small amounts. Consequently, in patients where PCWP is significantly lower than the LVEDP, it is unlikely that there is significant pulmonary return that traverses the pulmonary circuit. Thus, the PCWP may be significantly lower than the LVEDP. Moreover, patients may have a relatively reduced LV compliance compared to left atrial compliance.

There were also multiple instances wherein the LVEDP was significantly lower than the PCWP. We hypothesize that this discrepancy may be due to significant lung injury and the resultant intrapleural pressure elevations in these patients. The elevated intrapleural pressures may lead to significant atrial compression, leading to elevated atrial pressures that are transmitted to the pulmonary capillaries. In contrast, the thicker-walled LV may be less prone to compression, thus leading to a relatively lower elevation in the LVEDP (13). Given that severe lung injury occurs in patients with prolonged resuscitation requiring ECPR, this may be a common source of discordance. Moreover, a reduction in atrial compliance, either due to the index OHCA or pre-morbid cardiac conditions, may also lead to a relatively higher PCWP than LVEDP, as is seen in the heart failure with preserved ejection fraction population (14). Otherwise, none of the patients had mitral stenosis, pulmonary vein stenosis, or pulmonary veno-occlusive disease during the course of their hospitalization. In patients with severe mitral regurgitation, overestimation of the PCWP due to high V waves may also occur. Similarly, patients with atrial fibrillation or increased LV afterload may also exhibit reduced atrial compliance and severely elevated V waves. However, none of the patients in this case series had severe mitral regurgitation or exhibited atrial fibrillation at the time of the invasive hemodynamic study.

We also found that the PCWP and LVEDP appeared to be better correlated at the lowest level of V-A ECMO flow. Based on the above physiologic explanations, this may be because the reduction in the level of V-A ECMO flow allowed increased transpulmonary flow, thereby raising PCWP to a level that was more similar to LVEDP. Moreover, increased flow the left atrium may have allowed for the PCWP and LVEDP to be more similar.

Prior simulation data suggest that there are elevations of PCWP soon after the initiation of V-A ECMO (15–17). In these cases, PCWP is assumed to reflect LVEDP. Further, it is assumed that the elevated afterload leads to elevations of LVEDP and this is transmitted backwards into the pulmonary vasculature. Our data suggest that this may not be universally evident. This has also been proposed in the human literature. Schrage et al. reported a significant and immediate rise in PCWP in three patients that had V-A ECMO placement. This was used as a justification to implement LV unloading strategies. While the PCWP did decline soon after implementation of the LV unloading strategy, LVEDP was not measured in these patients.

Our findings may have clinical implications. Like other ambulatory and chronic cardiovascular conditions where there may be discrepancies in LVEDP and PCWP, the hemodynamic management of V-A ECMO may warrant separate evaluation of LVEDP and PCWP. This may be particularly pertinent at important junctures in the clinical care of these patients, such as when deciding whether unloading strategies are required. It may be particularly helpful to evaluate both PCWP and LVEDP at varying levels of flow, should the patient be able to tolerate that.

Our study has several limitations. Our case series carries all of the limitations that a small number of observations may be expected to carry. Specifically, there is likely a vast spectrum of hemodynamic phenotypes that may exist and only a small portion are represented here. There may be heterogeneity in the reduction of flows as a proportion of total flow (cardiac output + V-A ECMO). Future studies should aim to evaluate a strictly protocolized turn down to similar levels of flow in homogenous cohorts. Our study is also prone to selection bias—only patients that were thought to be able to tolerate reduction in V-A ECMO flows as part of a turndown were included in our study. It is also important to note that the relationship between PCWP and LVEDP may be dynamic. Thus, the magnitude and directionality of the PCWP and LVEDP relationship may change over time during the V-A ECMO treatment course of a single patient. Given that our investigation only evaluated invasive left and right heart pressures once in each of the patients, we were unable to evaluate this. Finally, it is possible that the differences in PCWP and LVEDP were present due to human error. However, we meticulously zeroed the catheters before each V-A ECMO flow phase and did blinded, off-line reviews of saved hemodynamic waveforms in an effort to prevent bias and human error. Even still, some measurements are too discrepant to explain by human error alone.

## Conclusion

In this small case series, we demonstrate that there is an inconsistent correlation between the magnitude and directionality of PCWP and LVEDP. Clinicians who manage V-A ECMO patients should consider monitoring both separately to guide clinical management.

## Data Availability

The raw data supporting the conclusions of this article will be made available by the authors; all reasonable requests for data will be considered.
